# Local Anæsthetics

**Published:** 1898-09

**Authors:** 


Local Anæsthetics. As a local anæsthetic in the removal of small tumors, the opening up of fistulous tracts, etc., a mixture consisting of antipyrine, 2 parts; cocaine, 1 part, and water, 10

parts, is highly recommended. A 50 per cent. solution of antipyrine alone may also be used for this purpose.

